# Illuminating the cells: transient transformation of citrus to study gene functions and organelle activities related to fruit quality

**DOI:** 10.1038/s41438-021-00611-1

**Published:** 2021-08-01

**Authors:** Jinli Gong, Zhen Tian, Xiaolu Qu, Qiunan Meng, Yajie Guan, Ping Liu, Chuanwu Chen, Xiuxin Deng, Wenwu Guo, Yunjiang Cheng, Pengwei Wang

**Affiliations:** 1 Key Laboratory of Horticultural Plant Biology (Ministry of Education), College of Horticulture and Forestry Science, Huazhong Agricultural University, 430070 Wuhan, China; 2 National R&D Centre for Citrus Preservation, Huazhong Agricultural University, 430070 Wuhan, China; 3 Interdisciplinary Sciences Research Institute, Huazhong Agricultural University, 430070 Wuhan, China; 4 Guangxi Academy of Specialty Crops/Guangxi Engineering Research Center of Citrus Breeding and Culture, 541004 Guilin, China

**Keywords:** Cell biology, Protein trafficking in plants, Biological techniques

## Abstract

Although multiple microscopic techniques have been applied to horticultural research, few studies of individual organelles in living fruit cells have been reported to date. In this paper, we established an efficient system for the transient transformation of citrus fruits using an *Agrobacterium*-mediated method. Kumquat (*Fortunella crassifolia* Swingle) was used; it exhibits higher transformation efficiency than all citrus fruits that have been tested and a prolonged-expression window. Fruits were transformed with fluorescent reporters, and confocal microscopy and live-cell imaging were used to study their localization and dynamics. Moreover, various pH sensors targeting different subcellular compartments were expressed, and the local pH environments in cells from different plant tissues were compared. The results indicated that vacuoles are most likely the main organelles that contribute to the low pH of citrus fruits. In summary, our method is effective for studying various membrane trafficking events, protein localization, and cell physiology in fruit and can provide new insight into fruit biology research.

## Introduction

Citrus is one of the most important and highest-yielding fruits in the world, and identifying genes associated with desirable traits is important for the sustainable development of citrus production. However, due to its long juvenile phase and diverse genetic background^1^, obtaining transgenic citrus plants is difficult and time-consuming. Therefore, developing an effective method for the transient transformation of citrus species is important for gene function characterizations and high-throughput screening. The *Agrobacterium*-mediated infiltration of tobacco leaves is a commonly used method to test gene function in vivo or study protein subcellular localization^2^. However, because of their heterogeneous expression, such a system is not ideal for studying genes that are specifically expressed in fruits. Although transient transformation techniques have been tested in fruits, such as strawberry^3,4^, apple^5,6^, and tomato^7^, none of these techniques proved to be suitable for studying subcellular activities and cell physiology.

Conventionally, cell biological studies in perennial woody plants and fruits have relied on squashing, sectioning, or enzyme-mediated degradation of the cell wall to gain access to the inner compartments. Therefore, conclusions are inevitably obtained from observations of fixed plant tissue, which sometimes does not reflect what truly occurs in planta. For example, the endomembrane compartment is constantly moving and remodeling. It contains the plasma membrane (PM), endoplasmic reticulum (ER), Golgi apparatus, and vacuoles, which play vital roles in protein secretion, storage, and degradation^8–10^. These functional compartments need to maintain a suitable environment; any alterations of their structure, redox potential, pH gradient, or lipid composition may lead to severe developmental defects and affect cell viability^11,12^.

However, most of the studies on plant cell biology have been carried out in model systems; little is known about horticultural plants and fruits, which have pleiotropic features that are absent in *Arabidopsis* and crop species. For example, the local pH of every organelle is tightly controlled and is critical to its function. Organelle pH can be measured in vivo using a GFP-derived ratiometric pH sensor (pHluorin) in tobacco and *Arabidopsis*^13,14^. For fruits, pH homeostasis not only affects the cell physiological environment but is also relevant to fruit acidity and quality. Despite its importance, little is known about the pH environment within most intracellular compartments of fruit cells. For example, fruits of nonacidic Faris lemon had average pH of 5.8–5.9, whereas those of acidic Faris lemon was 4.0, and those of Frost Lisbon lemon were 3.5–3.6^15^. Therefore, there must be specific pathways regulating pH in different fruit varieties. To understand this biological process, it is essential to measure and monitor local pH at the subcellular level.

Here, kumquat fruits (Fortunella crassifolia Swingle) were selected for *Agrobacterium*-mediated transient expression. The kumquat is closely related to most major citrus cultivars whose genomic information is widely available. It is also characterized by its high sugar content, thin skin, and relatively few juice cells; these characteristics provide a good environment for infiltration and infection by *Agrobacterium*. The feasibility of this method was demonstrated by expressing different organelle markers to study protein localization, as well as by expressing pHluorin-derived reporters to measure the pH of specific compartments of citrus cells. In addition, our results demonstrate that this technique is efficient not only for cell biology studies but also for the functional characterization of genes related to fruit physiology and quality.

## Results and discussion

### *Agrobacterium*-mediated transformation of citrus fruits

**Fig. 1:**
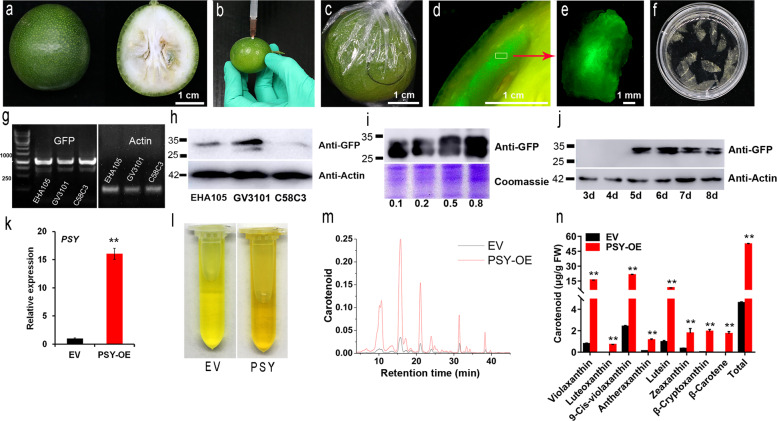
Schematic illustration of different steps in citrus fruit transient expression and detection. **a** A representative image of kumquat fruits used for injection. **b** Infiltration procedure: *Agrobacterium* solution (supplemented with p19) was injected into the epicarp at a depth of 0.1–0.3 cm. With gentle pressure on the plunger, the liquid spread into the fruit tissues. Each fruit was injected with ~0.4 ml *Agrobacterium* suspension. **c** Fruits were stored at room temperature for 2 days before being covered with cling film to prevent dehydration. **d**–**e** Dissected sections expressing GFP fusion proteins. **f** Cellulase treatment of citrus tissue for microscopic observation. **g**–**h** PCR and western blot analysis of GFP-HDEL expression in kumquat transformed with *Agrobacterium* strains EHA105, GV3101, and C58C3. **i** Western blot analysis of kumquat expressing GFP-HDEL at different bacterial (GV3101) densities. **j** Kumquat fruit expressing GFP-HDEL at 3 d, 4 d, 5 d, 6 d, 7 d, and 8 d after injection; actin was used as a reference. **k** Relative expression of the *PSY* gene in kumquat pulp after infiltration. EV, empty vector. **l**–**m** Carotenoid extracts were measured using pulp injected with EV (black) and *PSY* (red). **n** Carotenoid content in pulp infiltrated with *PSY* and EV. The data are presented as the mean ± SD of 3 independent replicates. Analysis of variance was performed to evaluate the significant differences based on the Student’s *t*-test at the significance levels of *P* < 0.05 (*) and *P* < 0.01 (**). Considering individual variation, all data presented here were generated from at least 15 fruits, with a minimum of 3 repeats

The transformation of citrus fruits was carried out in kumquat (Fig. 1a), which is one of the major citrus cultivars in southern China^16^. Kumquat blooms four times a year, and fresh fruits are available from November to March of the next year. With good preharvest management and postharvest storage conditions, the period of kumquat availability can be further extended. For the transformation experiment, fruits at the green ripening stage (~120–180 days after flowering) were used, and they were infiltrated with *Agrobacterium tumefaciens* carrying an expression vector (35S promoter-driven). The bacterial solution was gently injected into the epicarp at a depth of 0.1–0.3 cm (Fig. 1b). The mixture could distribute to an area with a radius of 0.8–1.3 cm from the injection point, as indicated using a red dye (Supplementary Fig. S1). The infiltrated fruits were stored at room temperature for ~2 days and wrapped with cling film to prevent dehydration (Fig. 1c). Approximately 5 days after injection, fluorescent signals were clearly visible near the site of injection (Fig. 1d–e). We tested this method using different citrus cultivars, including orange, pomelo, and mandarin (Supplementary Fig. S2) and found that kumquat constantly produced good signals and high transformation efficiency. In addition, fluorescent signals could be detected in different tissues of the kumquat, including the flavedo, albedo, juice sacs, and partition, into which the *Agrobacterium* solution penetrated (Supplementary Fig. S3).

Moreover, we tested different *Agrobacterium tumefaciens* strains (e.g., EHA105, GV3101, and C58C3), all of which carried a construct expressing GFP-HDEL (an ER marker). Western blot and PCR results demonstrated that GFP was expressed in all three experiments, and GV3101-mediated transformation produced the highest amount of protein (Fig. 1g–h). The optical density (OD_600_) of *Agrobacterium* also affects the level of protein expression (Fig. 1i), and it is necessary to optimize the best OD_600_ for a new construct before the actual study. If potential overexpression artifacts are a major concern, using the lowest OD_600_ that produces a strong enough signal is recommended. Otherwise, an OD_600_ of 0.5–0.8 can be used to provide maximum expression. Moreover, it is also important to keep the injection volume between 0.2 and 0.5 ml for each fruit, as overdoses of infiltration buffer may result in unexpected rotting. In most experiments, protein expression became detectable 4–5 days after infiltration (Fig. 1j). Protein expression could even be detected up to one month after infiltration if the fruits were kept under appropriate conditions (Supplementary Fig. S4). This prolonged-expression pattern was identified in most constructs in our study and could potentially provide a wide time window for certain downstream analyses.

### *Using* the transient expression method for gene characterization

Fruits may have unique metabolic pathways that are absent in other plant tissues. Therefore, an efficient assay to test the gene function of a particular metabolic pathway could be useful for high-throughput assays. Here, phytoene synthase (PSY), which regulates the most critical step in carotenoid synthesis^17,18^, was selected as an example. The results indicated that the transient overexpression of *PSY* resulted in significantly higher expression of *PSY* at the mRNA level compared to the control fruits (Fig. 1k), concomitant with carotenoid accumulation (Fig. 1l–n). In addition, with a protein tag (e.g., GFP, HA, Myc), immunoprecipitation (Co-IP) or chromatin immunoprecipitation (ChIP-seq) could be performed to screen possible interacting patterners of citrus protein in its native environment. This is one of the advantages of our system over tobacco expression.

### Live cell imaging of citrus fruit cells

The use of GFP and its derivatives is a landmark in cell biology; it can be fused with genes of interest to study their subcellular localization, dynamics, and protein–protein interactions^8^. To study subcellular activities and protein localization in living citrus fruits, infiltrated fruits were prescreened using a stereomicroscope to identify the successfully transformed area. Tissues with strong fluorescence signals were then hand-sliced into small pieces and transferred to a slide for observation. Alternatively, samples could be incubated in a buffer containing cellulose and macerozyme for 1–2 h to partially digest the cell wall (Fig. 1f). The main purpose of this additional step was to release the cells from the tissue to facilitate observation. Prolonged digestion can result in the production of protoplasts, but it is normally unnecessary; ideally, it is good to keep any disruptions of this process to a minimum. In addition, it is crucial to minimize mechanical damage and external pressure during the sectioning and imaging of fruit tissues.

**Fig. 2:**
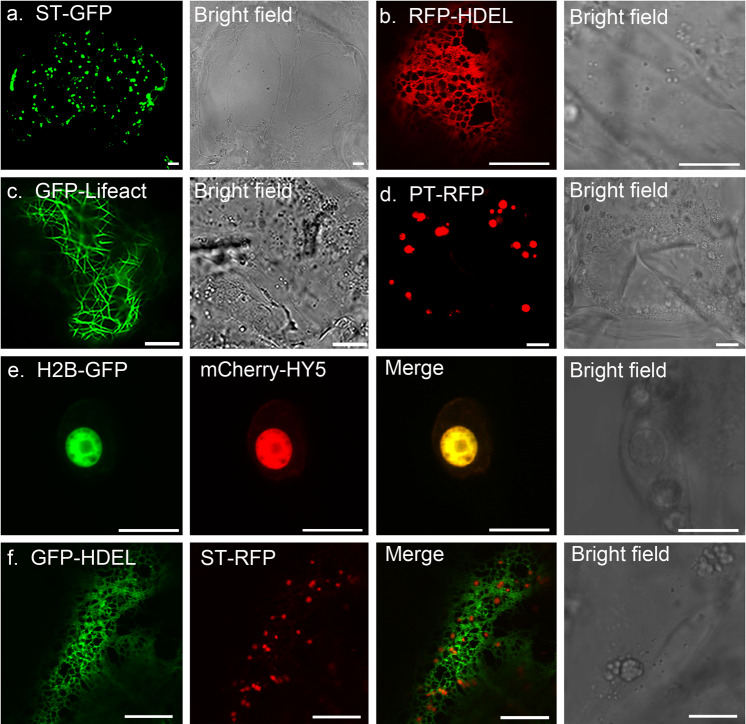
Transient expression of fluorescent fusion proteins in citrus fruit cells. All constructs were infiltrated at OD_600_ = 0.8, and images were taken 5 days after infiltration. **a**–**d** Representative images of fluorescent protein fusions localized to the Golgi apparatus (ST-GFP), ER (RFP-HDEL), actin cytoskeleton (GFP-Lifeact), and plastids (PT-RFP). **e** Transient expression of mCherry-HY5 (a transcription factor) localized to the nucleus that was colabeled with H2B-GFP (a nuclear marker). **f** Coexpression of GFP-HDEL and ST-RFP identified the close association between the ER network and Golgi bodies in kumquat fruit cells. Scale bar, 10 μm

Protein localization is one of the key factors involved in understanding protein function in vivo. Here, we infiltrated various organelle markers tagged with GFP/RFP, such as ST-GFP for the Golgi apparatus (Fig. 2a), RFP-HDEL for the ER network (Fig. 2b), GFP-Lifeact for the actin cytoskeletons (Fig. 2c), and PT-RFP for the plastids (Fig. 2d). Confocal microscopy suggested that their localization in citrus fruits was consistent with the results from *N. benthamiana* leaf epidermal cells (Supplementary Fig. S5). Next, we infiltrated different markers at the same time to study protein colocalization. For example, H2B-GFP and mCherry-HY5 (elongated hypocotyl5, a light-responsive transcription factor) were co-infiltrated and shown to be colocalized in the nucleus (Fig. 2e). Most organelles and membrane structures can physically interact with each other to form a nexus in eukaryotic cells^19,20^; one of the best examples of this is the ER-Golgi interface^21^. In our study, we coexpressed GFP-HDEL with ST-RFP to label the ER network and Golgi apparatus, and the results indicated a close association between these structures (Fig. 2f). This finding is consistent with other studies in higher plants^22^.

**Fig. 3:**
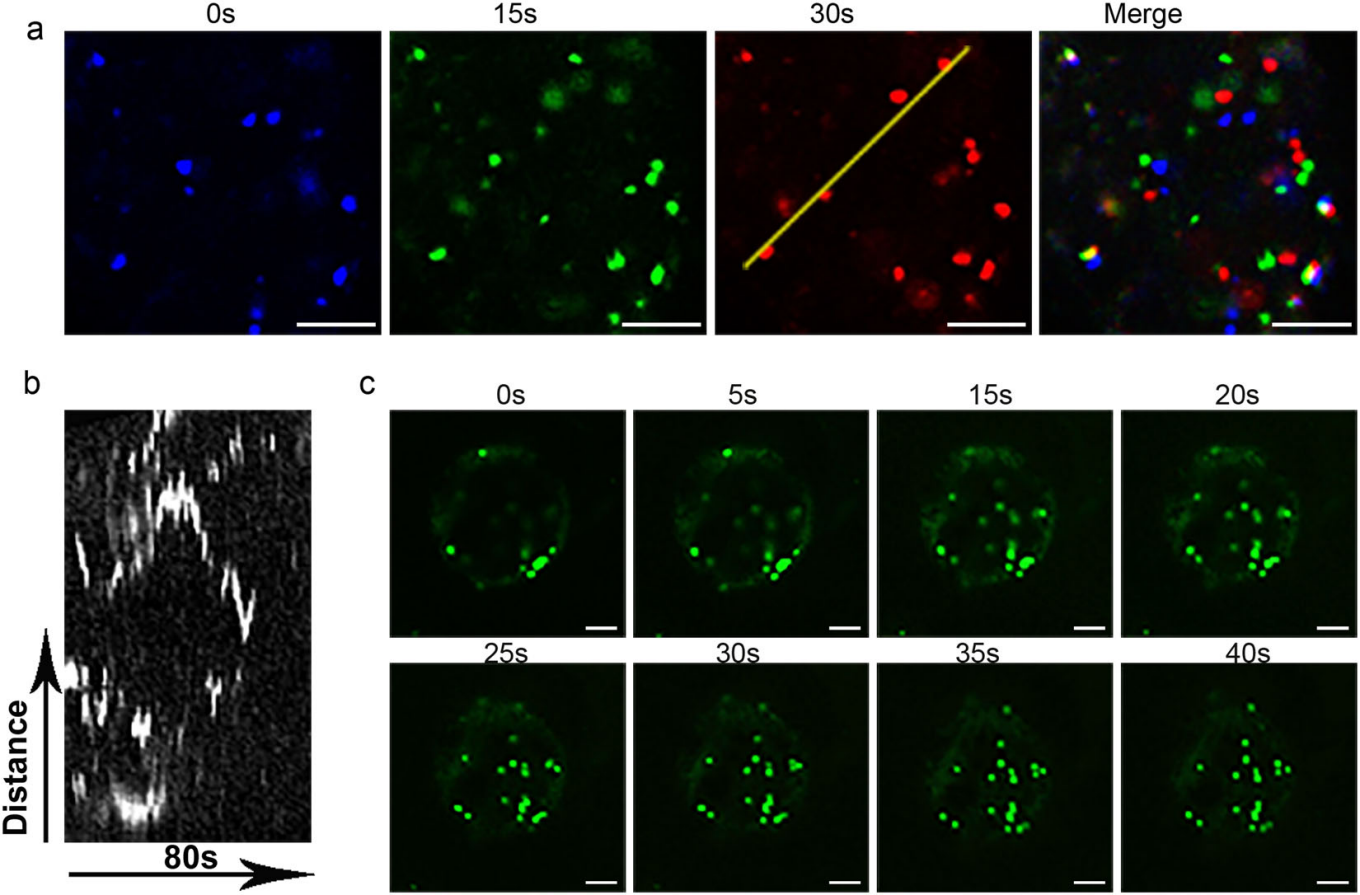
Live-cell imaging of ST-GFP reveals the dynamic movement of the Golgi bodies in citrus fruit cells. **a** Time series of ST-GFP-labeled Golgi bodies over 30 s. The images representing the different time points are pseudocolored: blue (0 s), green (15 s), and red (30 s). The merged picture with separated signals indicates that the puncta are mobile. **b** A kymograph was generated along the yellow line, demonstrating the movement of these structures. **c** Time-lapse images of ST-GFP-labeled Golgi bodies over 40 s in kumquat cells. Scale bar, 5 μm

It is worth pointing out that the structures of the ER and actin cytoskeleton are normally sensitive to mechanical disruption. Their structures remained intact in our experiment, indicating that the manipulation throughout the process caused little disruption to the cells. The Golgi apparatus is the compartment that regulates protein sorting and secretion^23^. In our study, ST-GFP-labeled Golgi bodies exhibited rapid movement, as indicated by our time series images and kymograph (Fig. 3). This result further indicated that live-cell imaging and advanced light microscopy techniques (e.g., BiFC, FRAP, FRET, and ratio imaging) could potentially be applied to study subcellar activities related to fruit physiology.

### *pHluorin*-derived fluorescence ratio imaging demonstrates the local pH environment at the organelle level

The total acid level is an important measure of fruit quality. As the fruit matures, the acidity of the juice increases, and the pH decreases. Recent developments in pH-sensitive fluorescent sensors have provided tools to measure the local pH of different subcellular compartments in vivo^13^. For example, pHluorin is a GFP derivative that can be activated differentially at 405 nm and 488 nm lasers depending on the surrounding proton concentration. A higher 405/488 ratio is expected under basic conditions, while a lower 405/488 ratio indicates a more acidic environment. In this study, we used this method to measure the local pH environment of the apoplast (PM-Apo), cytoplasm (Cyto-pH), ER (pH-HDEL) network, and vacuole (BCECF-AM).

**Fig. 4:**
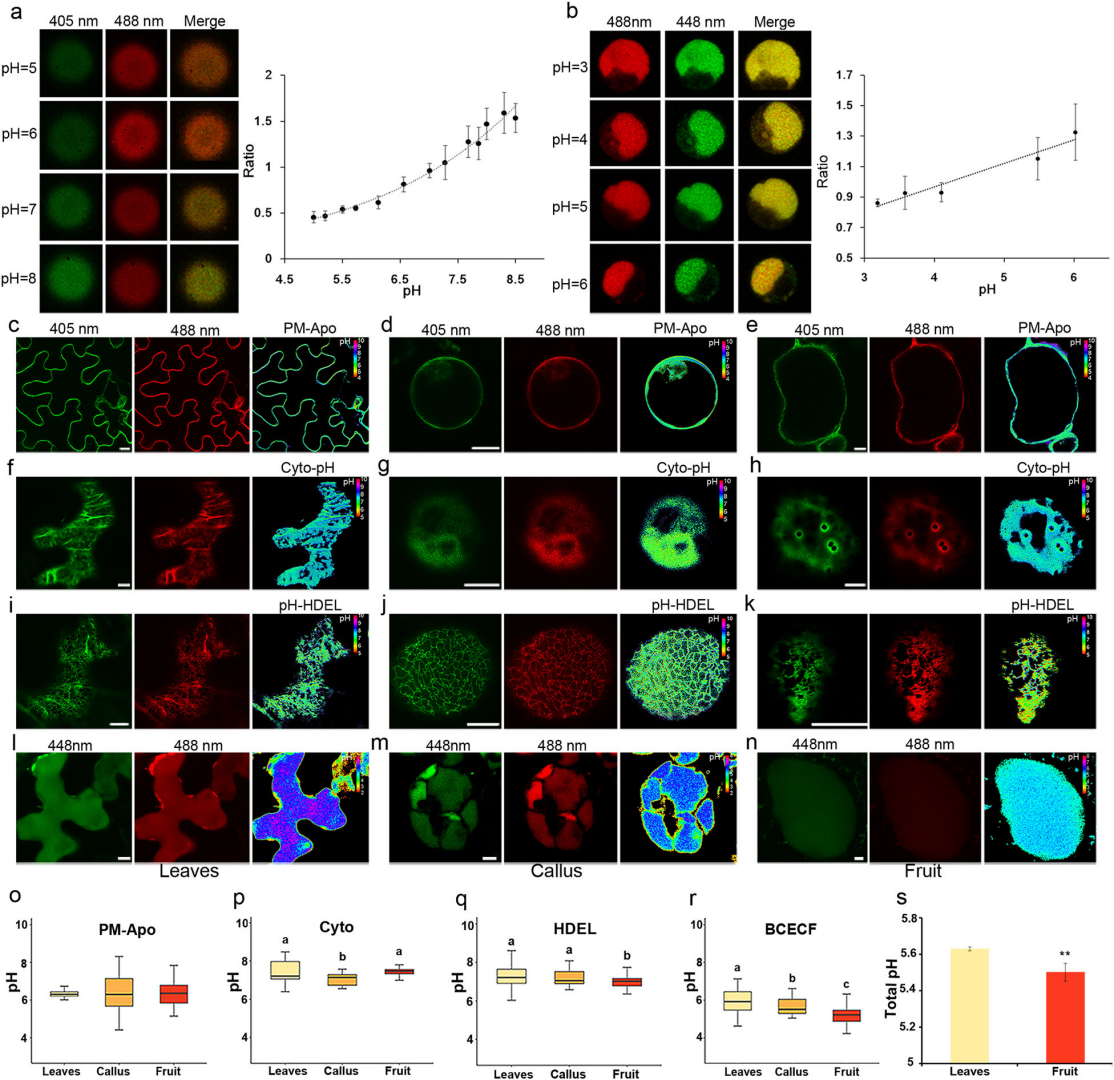
Measurement of the local pH value of different endomembrane compartments. Measurement of pH was performed in *N. benthamiana* leaf epidermal cells, citrus callus, and kumquat fruit cells transiently expressing pHluorin sensors (PM-Apo, PM facing the apoplast side, Cyto-pH for cytoplasm, pH-HDEL for ER), and the dye BCECF-AM for the vacuole. **a** In vitro calibration of pHluorin. Fluorescence emission ratios (405 nm/488 nm) of purified pHluorin protein in a buffer with a calibrated pH between 5.0 and 8.5. The equation applied for curve fitting is *y* = 0.0644e^0.3827x^, with *R*² = 0.9895. **b** Images of BCECF-AM-labeled vacuoles in various designated pH buffers. The calibration curve for BCECF-AM in a pH range from 3 to 6 was fitted using the equation *y* = 0.1542x + 0.3505, with *R*² = 0.9477. **c**–**e** Representative image of PM-Apo in *N. benthamiana* leaves (**c**), citrus callus (**d**), and kumquat fruits (**e**). **f**–**h** Transient expression of Cyto-pH in *N. benthamiana* leaves (**f**), citrus callus (**j**) and kumquat fruits (**h**). **i**–**k** Transient expression of pH-HDEL in *N. benthamiana* leaves (**i**), citrus callus (**j**), and kumquat fruits (**k**). All images in **c**–**k** were taken with the same confocal settings as the image (**a**). Scale bars, 10 μm. **l**–**n** Representative images of BCECF-AM staining of the vacuole in *N. benthamiana* leaves (**l**), citrus callus (**m**), and kumquat fruits (**n**). Images were taken using the same confocal settings as the image (**b**). Scale bars, 10 μm. **o**–**r** Comparison of the measured pH of PM-Apo (**o**), cytoplasm (**p**), ER (**q**), and vacuole (**r**) in *N. benthamiana* leaf cells, citrus callus, and kumquat fruit cells. PM-Apo, pH = 6.4 ± 0.2, 6.4 ± 1.0, 6.4 ± 0.6 in *N. benthamiana* leaf cells, citrus callus and kumquat fruit cells, respectively; Cyto, pH = 7.4 ± 0.5, 7.1 ± 0.3, 7.4 ± 0.2 in *N. benthamiana* leaf cells, citrus callus and kumquat fruit cells, respectively; ER, pH = 7.3 ± 0.5, 7.2 ± 0.4, 7.0 ± 0.3 in *N. benthamiana* leaf cells, citrus callus and kumquat fruit cells, respectively; Vacuole, pH = 6.0 ± 0.6, 5.7 ± 0.4, 5.2 ± 0.4 in *N. benthamiana* leaf cells, citrus callus, and kumquat fruit cells, respectively; *n* > 50; Significant differences were calculated using the Duncan test, *α* = 0.05. **s** Comparison of the total pH of crude extracts in *N. benthamiana* leaves and kumquat fruit. Student’s *t*-test at *t*he significance level of *P* < 0.01 (**)

First, His-tagged pHluorin was expressed in *E. coli* and purified using nickel-agarose beads under nondenaturing conditions. These agarose beads (attached to the pHluorin protein) were used for calibration at a specific pH, and the fluorescence emission ratio at 405/488 nm was measured. A series of measurements were performed under different pH conditions, and the data were plotted against pH as a sigmoidal curve^24^ (Boltzmann function, Fig. 4a).

The generated sigmoidal curve was then used to calculate the intracellular pH in the following experiments. However, pHluorin is not ideal for measuring any pH below 5, as its signal tends to be quenched under extremely acidic conditions. To overcome this problem, another fluorescent dye, BCECF-AM, was used in addition to measuring the pH within the vacuole. Using various pH buffers (pH = 3–6), the calibration curve was calculated as the fluorescence ratio of BCECF-AM at 488 nm/448 nm (Fig. 4b).

Before applying these pH sensors to citrus, we verified their subcellular localization in *N. benthamiana* leaves and measured their pH environment as described by Martinière et al.^13^. Using our calibration curves, the pH of the apoplast, cytoplasm, ER network, and vacuole in leaf cells was measured to be 6.4 ± 0.2, 7.4 ± 0.5, 7.3 ± 0.5, and 6.0 ± 0.6, respectively (Fig. 4). These results are very similar to those in previous studies, indicating that this system works well.

Next, the same studies were performed in citrus. Fruits were transformed as mentioned previously, while citrus calli were transformed using protoplasts by a PEG-mediated method. Surprisingly, the measured pH showed a similar value for the apoplast and small differences in the cytoplasm and ER lumen among leaves, calli, and fruit (Fig. 4). Since significantly different pH values were found in crude tissue extracts (Fig. 4s), we suspect that this variation may have been caused by the vacuole. To test this hypothesis, the vacuoles were stained with BCECF-AM, and the luminal measurements showed that kumquat fruits exhibited the lowest vacuolar pH (5.2 ± 0.4), significantly lower than the 5.7 in the callus and the 6.0 in *N. benthamiana* leaves (Fig. 4r).

**Fig. 5:**
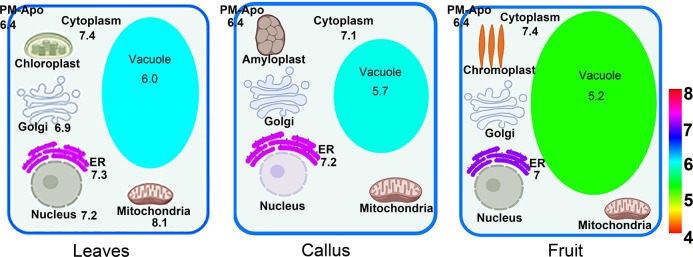
Comparison of the pH values of intracellular compartments in leaves, calli, and fruit cells. The pH values for the mitochondria, nucleus and Golgi in tobacco leaves were reported previously, and the other pH measurements were generated from this study

The unique pH value of each compartment is essential for protein sorting, as the binding or loading of cargo may rely on the formation of hydrogen bonds with their receptors, and this process is pH sensitive. Our results show that every organelle in the different plants has a signature pH (Fig. 5). Most compartments in fruit cells exhibit pH values similar to those of leaves and calli; however, pH variation is mainly observed in vacuoles. It is known that the pH value decreases along the membrane trafficking pathway (ER-Golgi-PVC/MVB-Vacuole); proton pumps that differentially localize to these compartments may work together to achieve such a pH gradient. However, for fruit varieties with high acid contents, unique vacuolar proton pumps or vacuole biogenesis pathways must be available to generate high proton gradients and create an extremely low pH environment^25^.

In summary, we studied citrus fruit cells at the subcellular level by using a series of fluorescent organelle markers and pH reporters. Different sensors (e.g., roGFP for redox potential^26^) could also be applied to study the communication between distinct intracellular compartments and specific metabolite (e.g., citrate^27^) accumulation at the organelle level in fruit cells. Future studies using this method could potentially be useful to reveal the function and regulatory mechanisms of a particular organelle during fruit development or postharvest.

## Materials and methods

### Plant material

Kumquat (*F. crassifolia* Swingle) fruits were collected from Rong’an and Guilin city, Guangxi Province, China. They were sterilized with 2% sodium hypochlorite solution for 2 min and rinsed thoroughly in water before *Agrobacterium* injection.

### Plasmid construction and Agrobacterium infiltration

RNA was extracted from citrus fruits using TRIzol reagent according to the HiPure HP Plant RNA Mini Kit (Magen), and single-stranded cDNA was synthesized by a cDNA Synthesis Kit (Ferment). The *PSY* and *HY5* coding sequences were PCR amplified from the cDNA library of kumquat fruit and cloned using the pCR™8/GW/TOPO TA Cloning Kit (Invitrogen). Recombinase reaction was performed using Gateway LR Clonase II Enzyme Mix (Invitrogen) according to the manufacturer’s instructions in order to insert the gene into the pMDC43 plasmid for overexpression.

*Agrobacterium* transformed with a gene construct was grown overnight in liquid LB medium to an O.D. of 0.8–1.0. Then, they were resuspended to a final O.D. of 0.8 in liquid injection medium containing 0.05 M MES, 2 mM Na_3_PO_4_, 0.5% (m/v) D-glucose, and 0.1 mM acetylsyringone.

### Analysis of gene and protein expression

Quantitative analysis of *PSY* expression levels was performed with a Roche LightCycler 480 system using the 2× LightCycler 480 SYBR Green master mix (Roche) and a three-step program. The primers were listed in Supplemental Table 1. A total amount of 30 μg protein was used for western blot analysis and detected by either GFP or RFP antibodies (Biorbyt) at a dilution of 1:5000 or 1:10,000, respectively. Goat anti-mouse HRP (Biorbyt) was used as the secondary antibody at a dilution ratio of 1:10,000. The fluorescent signals were detected using a chemiluminescent gel imaging system (Amersham Imager 600).

### HPLC analysis for carotenoid metabolites

Carotenoid extraction and quantitation were performed as described previously^28^. Carotenoid metabolites were analyzed by high-performance liquid chromatography (HPLC), which was performed in a Waters liquid chromatography system as described previously^29^. The carotenoids were identified by their characteristic absorption spectra and retention times based on the literature and standards purchased from CaroteNature (Lupsingen, Switzerland). The carotenoids were quantified using calibration curves at 450 nm.

### Light microscopy

Citrus tissues injected with the *Agrobacterium* suspension were gently placed onto microscope slides and immediately observed with a stereomicroscope (SZX7; Olympus) equipped with a DP70 camera. For confocal microscopy, a disposable sharp blade was used to quickly cut the expressed tissue as thinly as possible on microscope slides dipped in water droplets for direct observation. To avoid damage to fruit cells during ultrathin sectioning, alternatively, the samples were digested for ~1–2 h in a filter-sterilized enzymatic solution containing 1.5% cellulase R10, 0.4% macerozyme R1, 0.4 M mannitol, 20 mM KCl, 20 mM MES (pH = 5.8), and 10 mM CaCl_2_. The samples were mounted on microscope slides, very carefully sealed with nail polish, and imaged as described previously^30^. All fluorescent protein markers used in this study are listed in Supplemental Table 2.

### Protoplast preparation and transfection

*Citrus unshiu* callus “Guoqing No. 1” grown for ~20 days was selected and carefully clamped into a vessel containing the enzymatic solution (same as above) with tweezers. Then, enzymatic hydrolysis was induced by shaking the mixture slightly overnight under dark conditions to obtain enough protoplasts. Protoplast transfection was performed as recommended by Yoo et al.^31^.

### Calibration of pH sensors

*Escherichia coli* (BL21) was grown to OD_600_ = 0.8 at 37 °C, and 0.1 mM IPTG was added to the bacterial solution to induce the expression of the target protein overnight. Then, the bacteria were collected by centrifugation, washed three times with water, and sonicated in phosphate buffer. The bacterial lysate was centrifuged at 1000 rpm and 4 °C for 1 h, and the supernatant was collected and filtered through a 0.45 μm filter. Next, the target protein was purified using Ni-NTA His Bind Resin following the manufacturer’s instructions (Biotech, Wuhan, China). The recombinant pHluorin was diluted in 50 mM MES-KOH buffers with different pH values. Using a confocal microscope (SP8; Leica), fluorescence signals at emission wavelengths of 505–550 nm were collected with excitation wavelengths of 405 or 488 nm. The pH profile was expressed by converting the grayscale ratio image into pseudocolor using ImageJ. All data are shown as the means and standard deviations from at least 10 images for each indicated pH value. All curve fittings were performed using Origin 9.0 SR1(OriginLab Corp., Northhampton, MA, USA).

### Measurement of pH

To measure the pH of the specific pHluorin-targeted compartment, samples were excited at 405 or 488 nm as described above. The parameters of the images taken with CLSM were described by Martinière et al.^13^, and the pH value was extrapolated from the sigmoidal function obtained from the calibration curve in vitro. The vacuolar pH was measured with the cell-permeant and pH-sensitive fluorescent dye BCECF-AM according to Tang et al.^32^ with a 505–550 nm emission bandwidth of the PMT and excitation at 488 or 448 nm. The total pH was calculated by measuring the juice of the leaf and fruit tissues with a pH meter (ST3100, OHAUS).

## Supplementary information

Supplementary Fig S1-S5 and table S1-S2

Figure S1

Figure S2

Figure S3

Figure S4

Figure S5

Supplementary Fig S1-S5 and table S1-S2
