# Supplementary material for: Illuminating the cells: transient transformation of citrus to study gene functions and organelle activities related to fruit quality
Source: Hortic Res. 2021 Aug 1;8:175. doi: 10.1038/s41438-021-00611-1 (PMC8325690; doi:10.1038/s41438-021-00611-1)
Supplement: Supplementary file 1 — Supplementary Fig S1-S5 and table S1-S2 [file 41438_2021_611_MOESM1_ESM.doc]

Supplementary figures for

**Illuminating the cells: transient transformation of citrus to study gene function and organelle activities related to fruit quality**

Jinli Gong^1,2,3#^, Zhen Tian^1,2,3#^, Xiaolu Qu^1^, Qiunan Meng^1,2,3^, Yajie Guan^1,2,3^, Ping Liu^4^, Chuanwu Chen^4^, Xiuxin Deng^1^, Wenwu Guo^1^, Yunjiang Cheng^1,2^ and Pengwei Wang^1,2,3^*

Institution addresses:

^1^Key Laboratory of Horticultural Plant Biology (Ministry of Education), College of Horticulture and Forestry Science, Huazhong Agricultural University, Wuhan 430070, China;

^2^National R&D Centre for Citrus Preservation, Huazhong Agricultural University, Wuhan 430070, China;

^3^Interdisciplinary Sciences Research Institute, Huazhong Agricultural University, Wuhan, 430070, China;

^4^Guangxi Academy of Specialty Crops/Guangxi Engineering Research Center of Citrus Breeding and Culture, Guilin, 541004.

*Corresponding author: Pengwei Wang ([wangpengwei@mail.hzau.edu.cn](mailto:wangpengwei@mail.hzau.edu.cn))

**
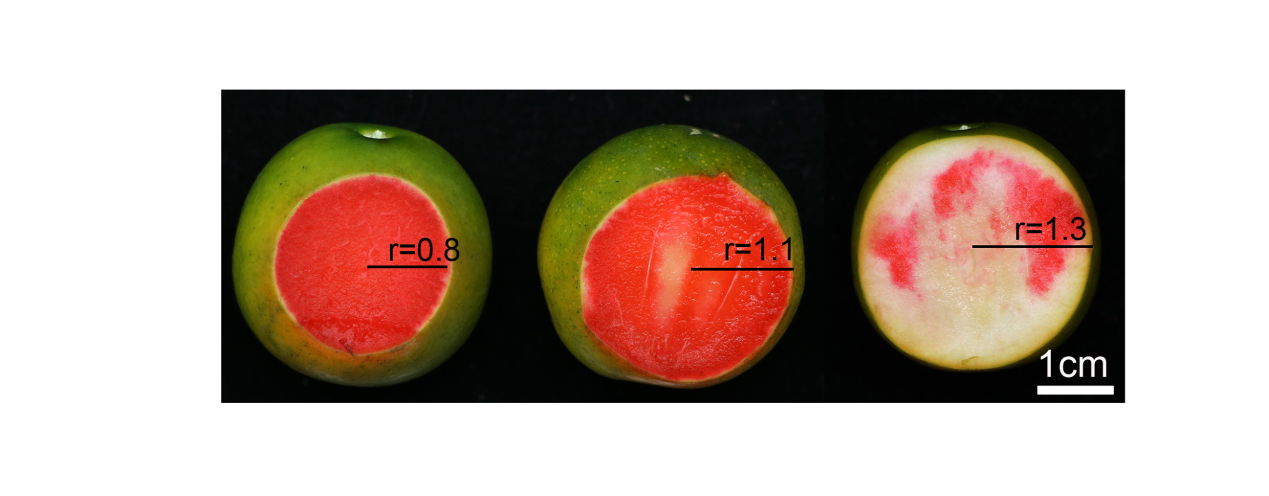
**

**Fig. S1 Citrus fruits were infiltrated with red ink to mimic the diffusion of bacteria solutions.**

**
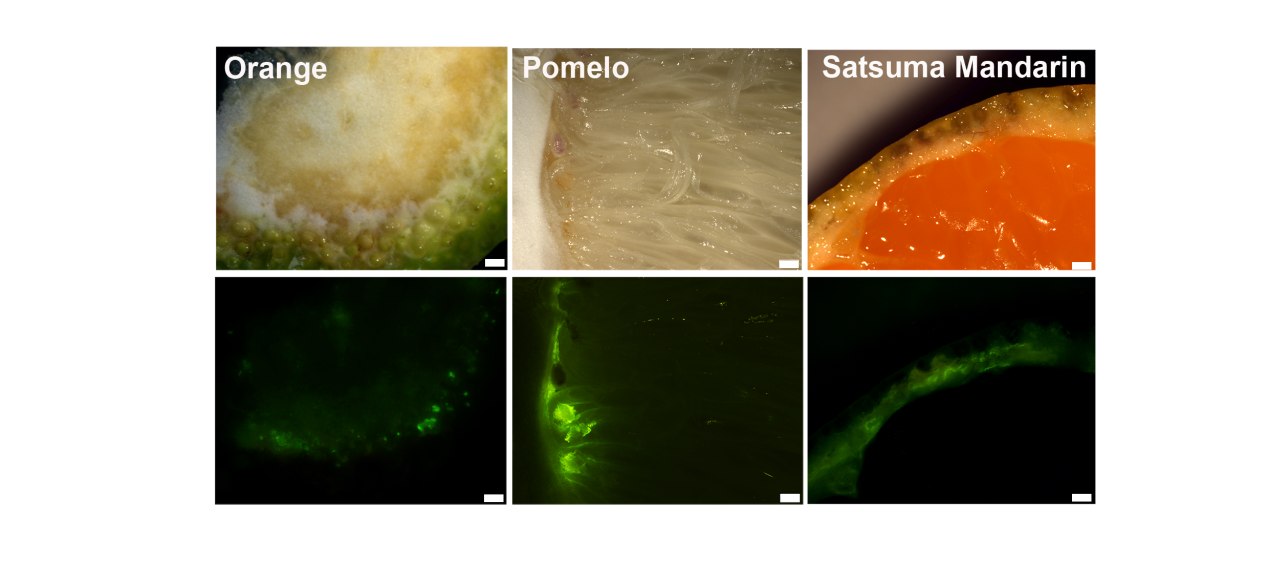
**

**Fig. S2** **Transient expression studies in different citrus varieties.** "Newhall" navel orange, pomelo and mandarin (on the tree, 150 DAF) were injected with GFP-HDEL, and kept for 15 day; then the transformed areas were observed using a bright field (above) and fluorescent microscope (below). Scale bar, 1000 μm.


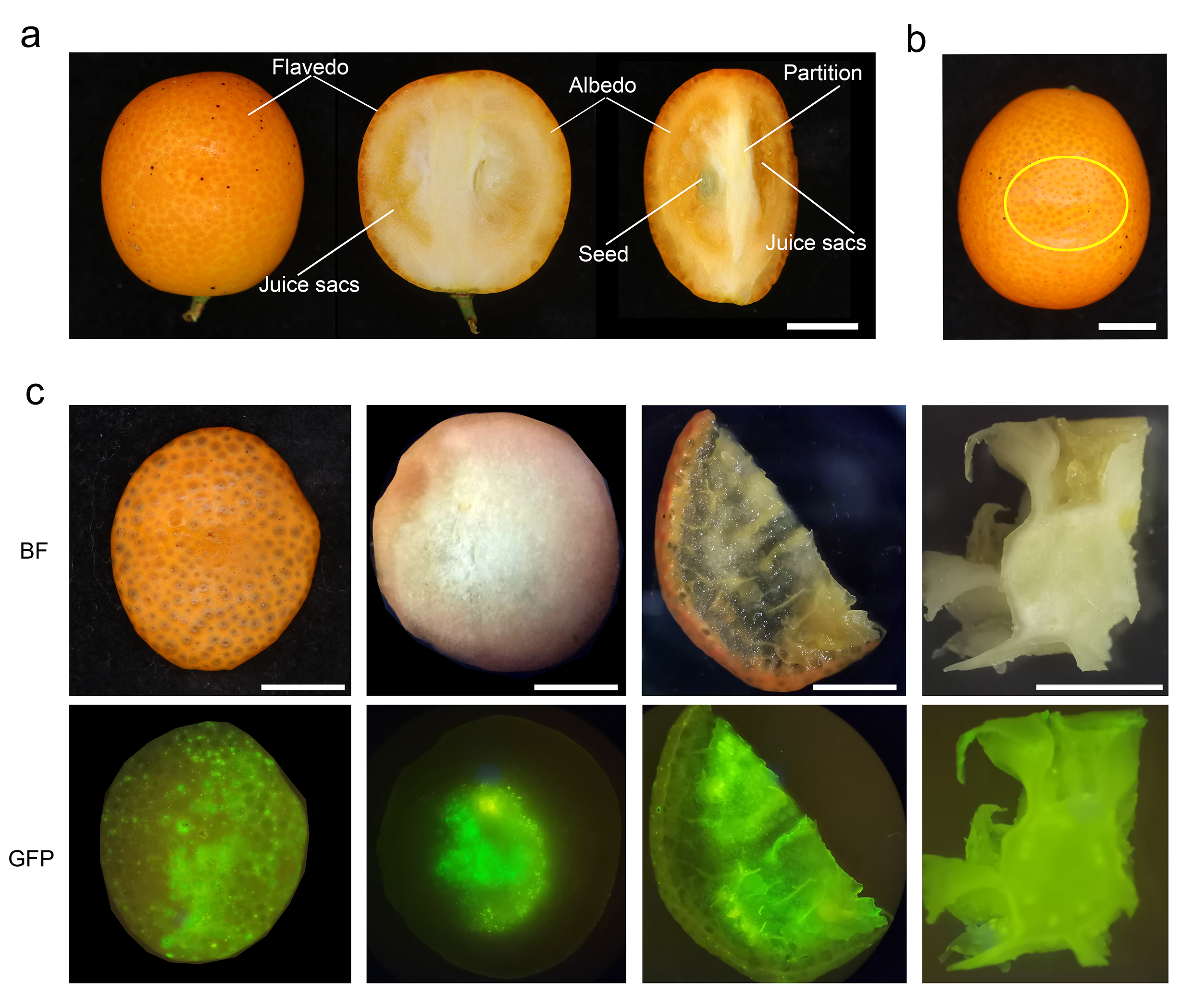
**Fig. S3 Validating the expression of transformed fusion protein in different tissues of kumquat. a** Anatomical view of the different tissues of the kumquat fruit. **b** A representative fruit for transient transformation. The yellow circle indicates the infiltrated area. **c** The expression of GFP-HDEL fluorescent protein in flavedo, albedo, juice sacs, and partition, respectively. BF, bright field. Scale bar, 1 cm.


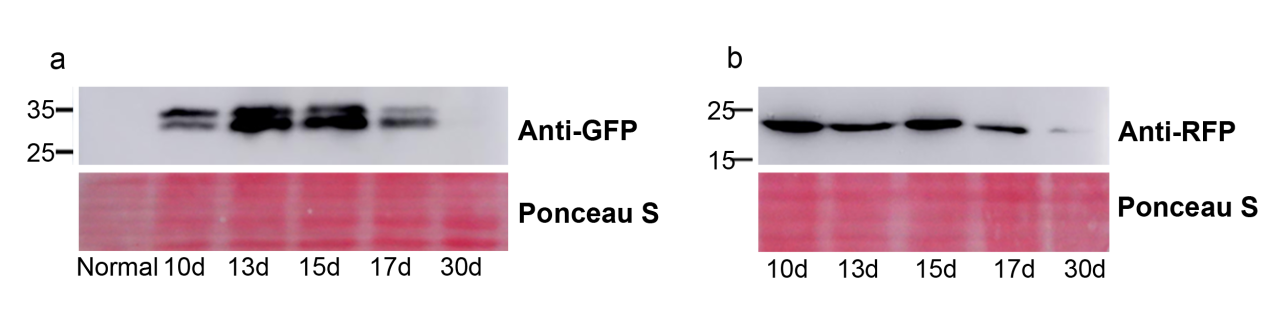


**Fig. S4 Validating the expression of transformed fusion protein using western blot. a-b** Western blotting analysis of GFP-HDEL and PT-RFP expression in kumquat 10 day to 30 days after injection, respectively. Blots were probed with GFP and RFP antibody, respectively. Normal, not injected.


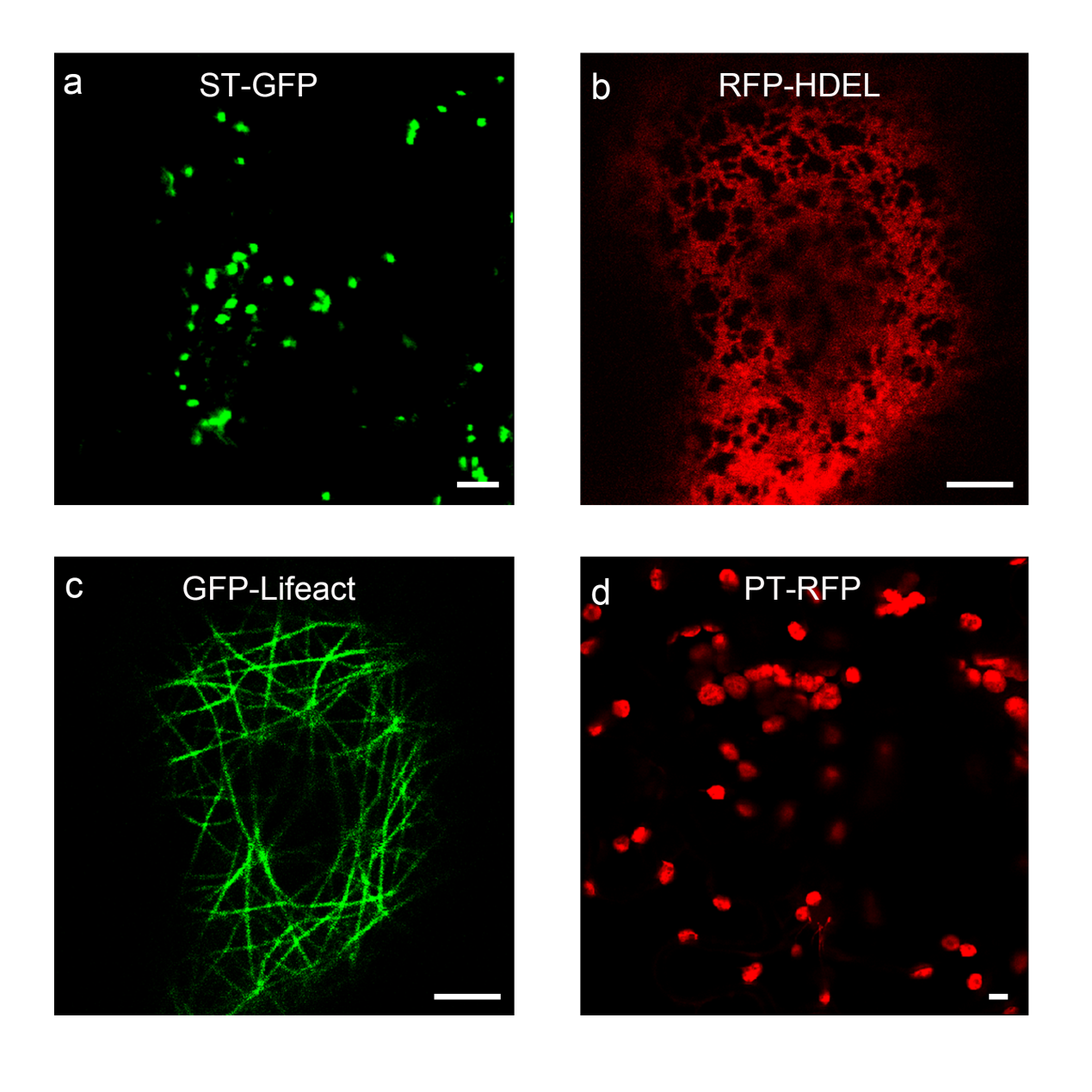


**Fig. S5 The expression of fluorescent organelle markers in *N. benthamiana* leaf epidermal cells. a-d** Representative images of fluoresenct protein labelled Golgi apparaturs (ST-GFP), ER (RFP-HDEL), actin cytoskeleton (GFP-Lifeact) and plastids (PT-RFP)**,** respectively. All constructs were infiltrated at OD_600_ = 0.1, and images were taken 3 days after infiltration. Scale bar, 5 μm.

**Table S1.** Primers used in this study

| Primer Name | | | Forward Primer Sequence | | Reverse Primer Sequence | | References | |
| --- | --- | --- | --- | --- | --- | --- | --- | --- |
| **Gene expression** | | | | | | | | |
| GFP | | | ATCCCACCCCTACTCCAAAAA | | CTCGATGTTGTGGCGGATC | |  | |
| Actin | | | CCAAGCAGCATGAAGATCAA | | ATCTGCTGGAAGGTGCTGAG | | Liu et al., 2007[^33^](#_ENREF_1) | |
| **Plasmid construction** | | | | | | | | |
| HY5 | aaaaagcaggctccATGCAAGAACAAGCAACGAG | | | | AGAAAGCTGGGTTTTAAAAGGACCCATCGGCAT | | |  |
| PSY | aaaaagcaggctccATGTCTGTTACATTGCTGTG | | | | AGAAAGCTGGGTCTTAAGCCTTACTGGTATATA | | |  |
| **RT-qPCR** | | | | | | | | |
| PSY | | CCCGGACTGCTGTGTTTAAT | | GAGCAAGGATGCCTCAAATC | | Liu et al., 2007[^33^](#_ENREF_1) | | |
| Actin | | CCAAGCAGCATGAAGATCAA | | ATCTGCTGGAAGGTGCTGAG | | Liu et al., 2007[^33^](#_ENREF_1) | | |

**Table S2. List of fluorescent protein markers used in this study**

| **Name** | **Details** | **Reference** |
| --- | --- | --- |
| **Subcellular localization** | | |
| ST-RFP | RFP marker for Golgi apparatus | Boevink et al*.,* 1998[^23^](#_ENREF_2) |
| ST-GFP | GFP marker for Golgi apparatus | Boevink et al*.,* 1998[^23^](#_ENREF_2) |
| RFP-HDEL | RFP marker for endoplasmic reticulum | Boevink et al*.,* 1998[^23^](#_ENREF_2) |
| GFP-HDEL | GFP marker for endoplasmic reticulum | Boevink et al*.,* 1998[^23^](#_ENREF_2) |
| GFP-Lifeact | GFP marker for the actin cytoskeleton | Smertenko et al*.,* 2010[^34^](#_ENREF_2) |
| PT-RFP | RFP marker for plastids | Nelson et al*. ,*2007[^35^](#_ENREF_3) |
| H2B-GFP | GFP marker for the nucleus | C[lone](../../../AppData/Local/youdao/dict/Application/7.2.0.0511/resultui/dict/) in this study |
| **Constructs for pH detection** | | |
| PM-Apo | A pHluorin anchored in PM facing the apoplasm | Martinière et al., 2018[^36^](#_ENREF_4) |
| Cyto-pH | pH Sensor targeting cytoplasm | Martinière et al., 2013[^13^](#_ENREF_4) |
| pH-HDEL | pH Sensor targeting ER | Martinière et al., 2013[^13^](#_ENREF_4) |
