# Supplementary figures and images for: Illuminating the cells: transient transformation of citrus to study gene functions and organelle activities related to fruit quality
Source: Hortic Res. 2021 Aug 1;8:175. doi: 10.1038/s41438-021-00611-1 (PMC8325690; doi:10.1038/s41438-021-00611-1)

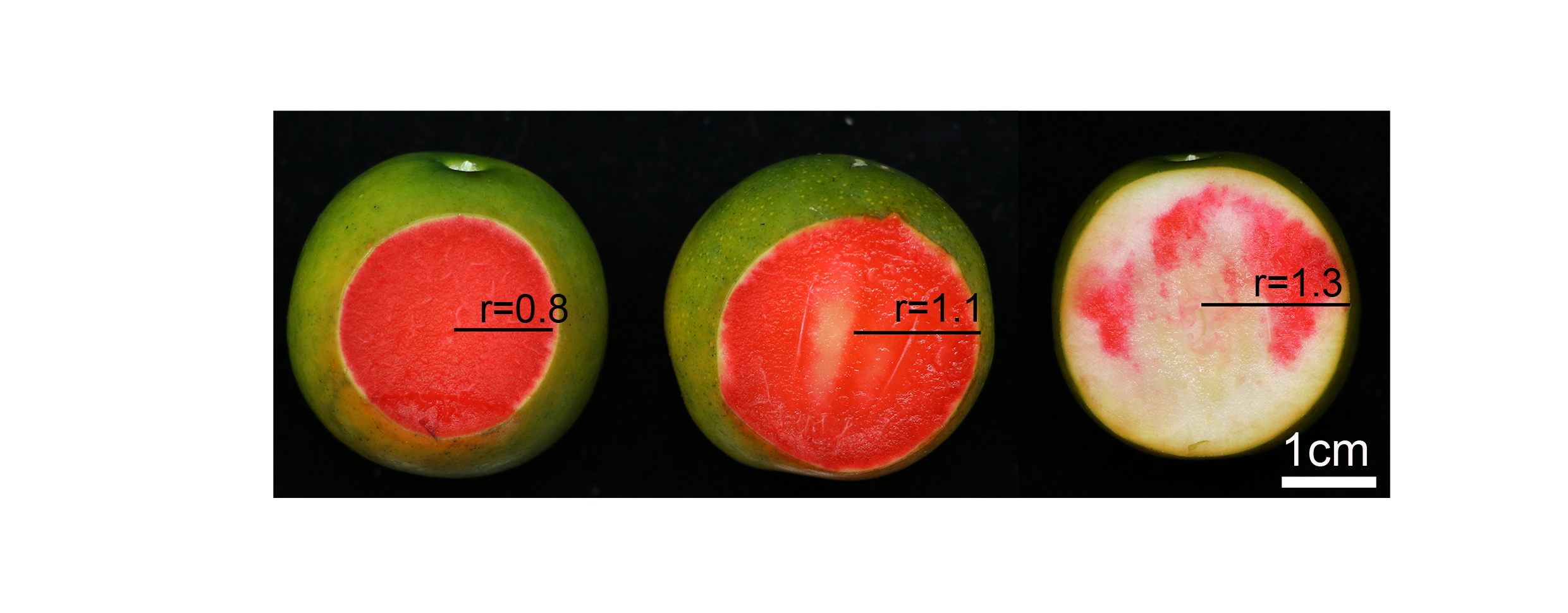

Supplement: Supplementary file 2 — Figure S1 [file 41438_2021_611_MOESM2_ESM.tif]

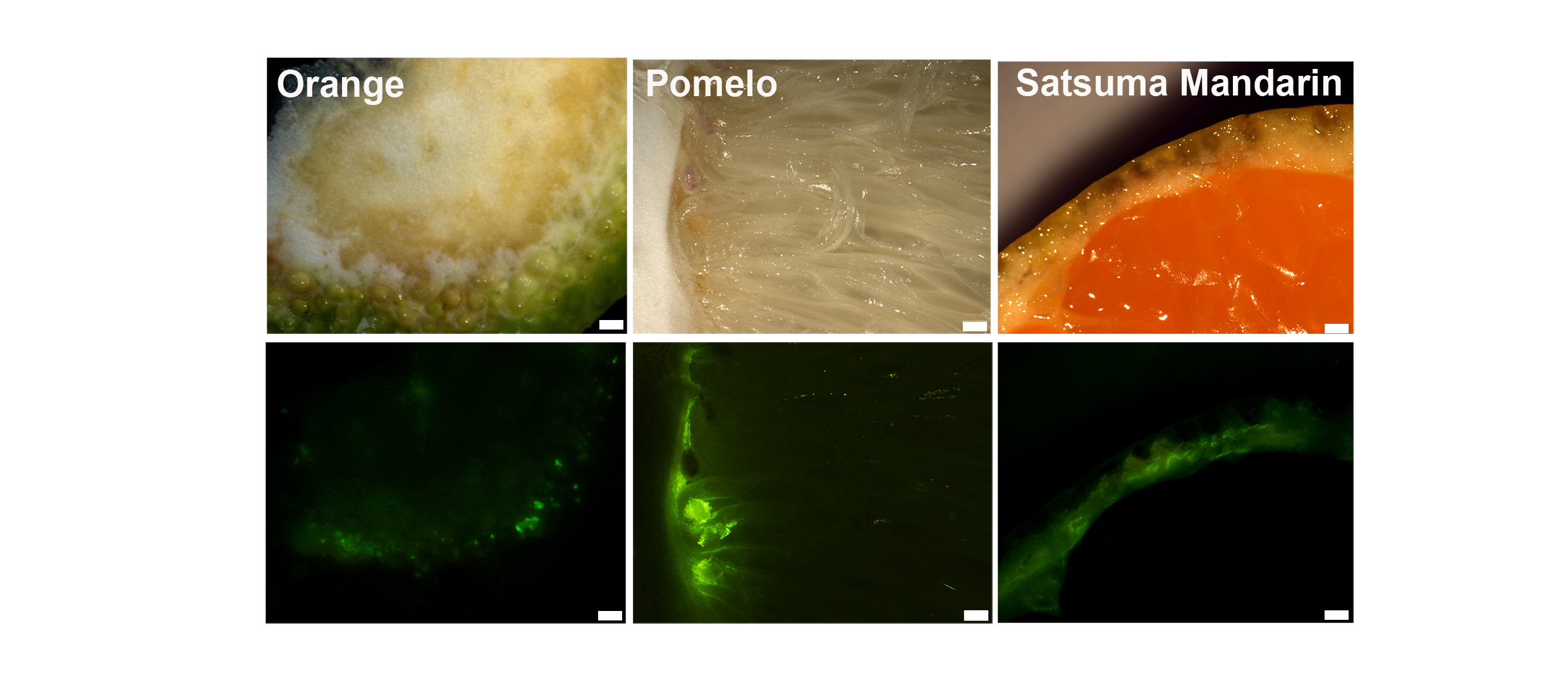

Supplement: Supplementary file 3 — Figure S2 [file 41438_2021_611_MOESM3_ESM.tif]

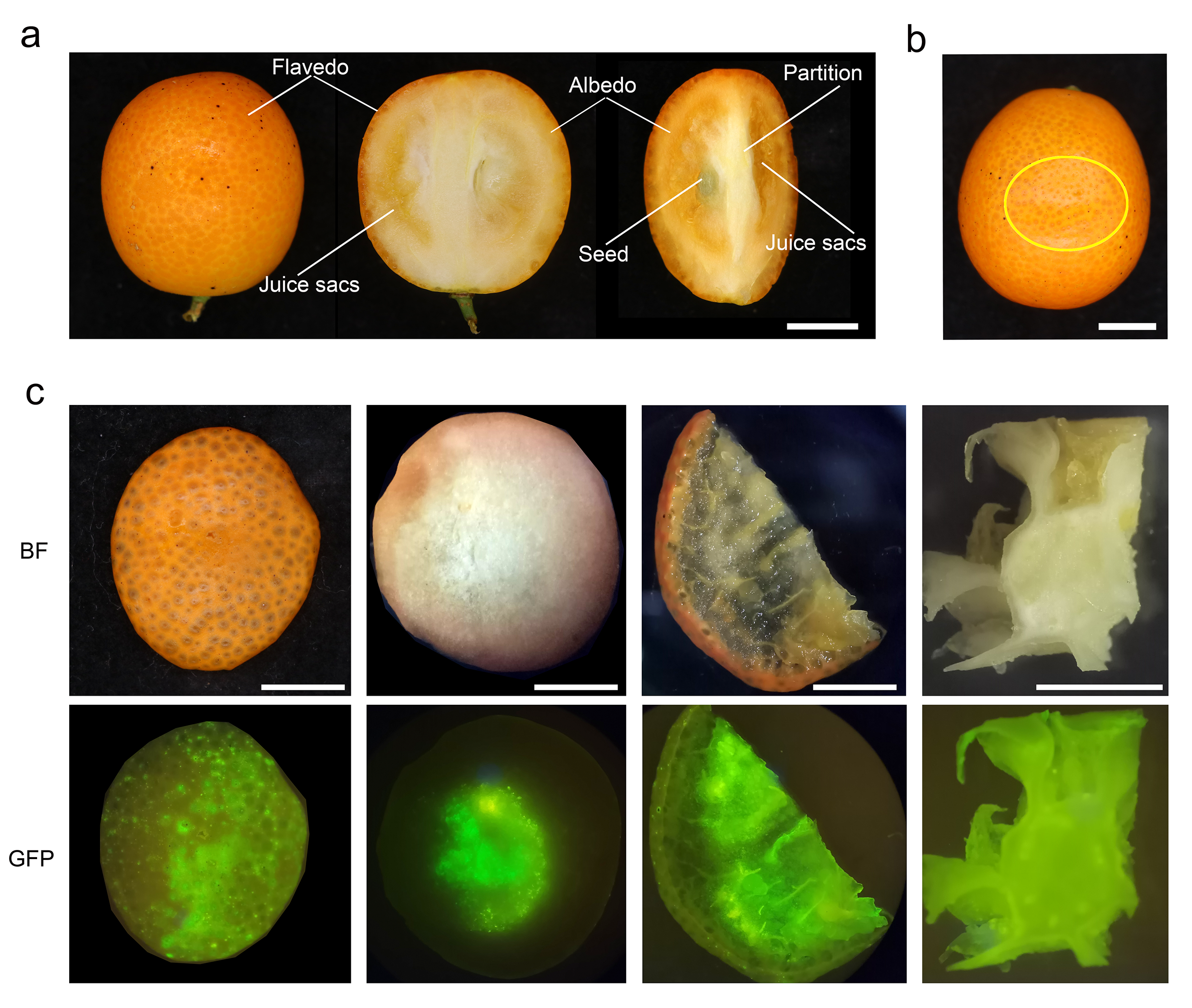

Supplement: Supplementary file 4 — Figure S3 [file 41438_2021_611_MOESM4_ESM.tif]

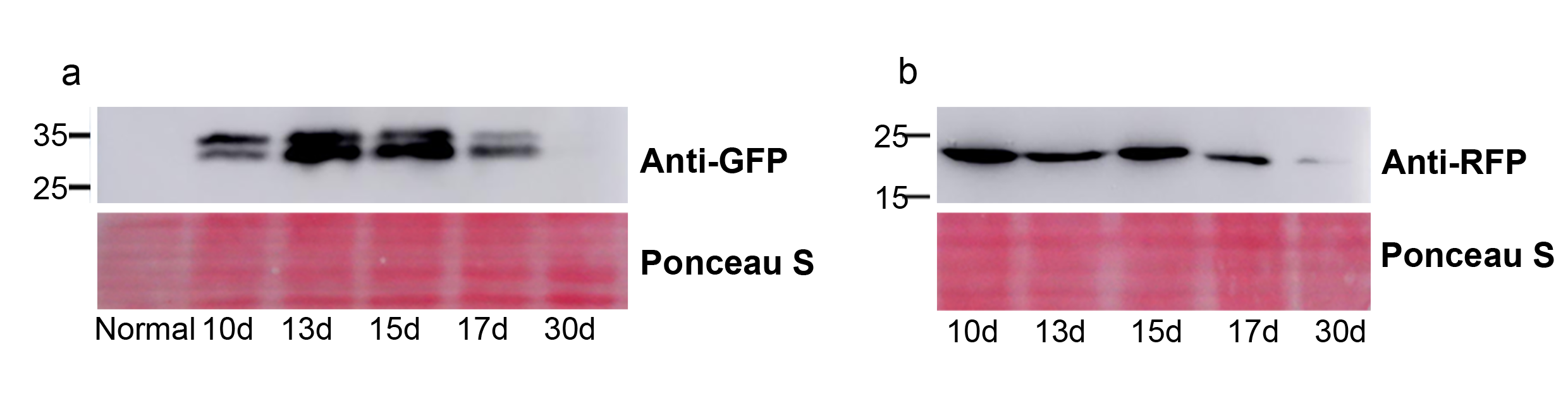

Supplement: Supplementary file 5 — Figure S4 [file 41438_2021_611_MOESM5_ESM.tif]

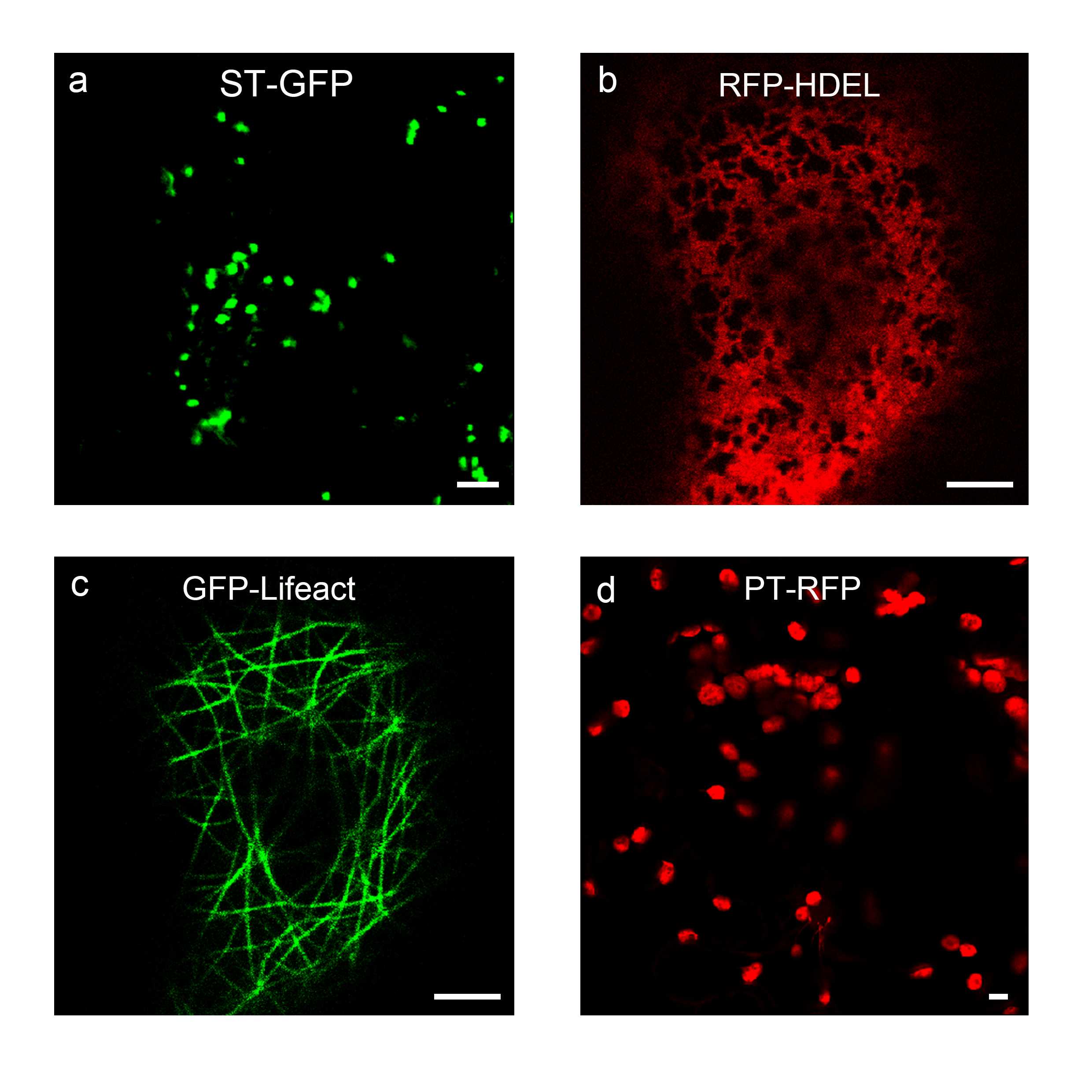

Supplement: Supplementary file 6 — Figure S5 [file 41438_2021_611_MOESM6_ESM.tif]
